# Intestinal Surgery

**Published:** 1904-07

**Authors:** Marshall Clinton

**Affiliations:** Buffalo, N. Y., Attending Surgeon, Sisters of Charity, and Erie County Hospitals, Buffalo, N. Y.; Instructor in Surgery in the University of Buffalo


### Intestinal Surgery.

### A Study of the Treatment of a General Spreading Peritonitis.

BY MARSHALL CLINTON, M. D., Buffalo, N. Y.,

Attending Surgeon, Sisters of Charity, and Erie County Hospitals, Buffalo, N. Y.; Instructor in Surgery in the University of Buffalo.

THERE is probably no subject on which a wider difference of opinion will be found among leading operators of today than in their treatment of a spreading peritonitis following perforation of a septic appendix. Individual opinion is generally based on clinical experience, but many men of equal experience hold opposite views of the proper treatment of this condition. Until this subject is settled one way or the other, every case which may shed light on the value or uselessness of each type of procedure should be added to the side where it belongs. The following case offers some interesting features:

On January 28, W. H., aged 20, felt a sudden, sharp pain in the right lower quadrant of his abdomen while lifting some timber. He left work, went home and was confined to his bed and under medical advice. The pain increased, his abdomen became distended on the following day and he began to vomit. There was a slight raise in temperature and a pulse running from 100 to 120. There was constipation and enemas were without result.

I was called to see the patient January 29, in the evening, and expressed the opinion that he would die unless an operation was performed at once. This was refused until twelve hours later when the condition of the patient became so serious that operation was accepted as the only chance for him.

The patient entered the hospital with a pulse of 140, temperature 96°, vomiting continued and he was extremely prostrated.

He was prepared for immediate operation. An incision over the appendix revealed a quantity of creamy, foul-smelling pus that seemed to fill the entire abdominal cavity. There were few adhesions and pus was found in every part. Openings were made in the middle line, on opposite side and in both flanks. Over ten gallons of hot salt solution were used to flush out his belly, and at the end of that irrigation his general condition seemed slightly improved and the abdominal cavity apparently clean. Large drainage tubes wrapped with gauze were introduced into each incision, draining most of the peritoneal cavity. Twelve hours later while lying in bed with the head of the bed raised to drain the abdomen by gravity he was again irrigated with a large amount of salt solution and a considerable quantity of the same creamy, foul pus was washed out of the tubes as found at the first irrigation.

The next day, or twenty-four hours later, the abdominal cavity was found clean on irrigation and the tubes all adherent. The patient convalesced rapidly and recovered completely except for a fecal fistula which had to be closed by a second operation on March 9. He was discharged from the hospital with instructions to watch for a possible obstruction developing and explicitly cautioned to watch for the point of obstruction should it occur.

On April 8, at 6 in the morning, the patient telephoned that he was in pain and wanted me at once. He had begun to vomit and said he had felt obstructed since 3 o'clock the previous afternoon. He designated the point of obstruction and wished to go to the hospital immediately. He was entered and prepared for operation. An incision over the point he had indicated along the outer border of the right rectus muscle showed the upper portion of the ileum constricted by an adhesion band. This was easily released with the finger and, milking the contents of the intestine into the empty lower bowel, the incision was closed tightly. This wound healed in ten days and the patient has been gaining in health and strength since his discharge from the hospital.

The interest in this case seems to be that the patient did well with wide and free drainage for the first few hours, and his peritoneum cleaned up after about twenty-four hours with copious irrigation. One would judge from the result of the second irrigation that it was a wise procedure, in that it removed a large amount of material which would have remained in his belly to be absorbed. Of course this class of cases is liable to develop an obstruction subsequent to operation from contraction of adhesions, but when the patient is warned and the nature of the trouble explained, there is generally little trouble in obtaining consent to early successful operation.

466 Franklin Street.

